# The effect of chronic kidney disease on tissue formation of *in situ* tissue-engineered vascular grafts

**DOI:** 10.1063/5.0138808

**Published:** 2023-05-23

**Authors:** Paul J. Besseling, Merle M. Krebber, Joost O. Fledderus, Martin Teraa, Krista den Ouden, Melanie van de Kaa, Petra M. de Bree, Aurelie Serrero, Carlijn V. C. Bouten, Patricia Y. W. Dankers, Martijn A. J. Cox, Marianne C. Verhaar

**Affiliations:** 1 Department of Nephrology and Hypertension, Regenerative Medicine Center, University Medical Center Utrecht, Utrecht, The Netherlands; 2 Department of Vascular Surgery, University Medical Center Utrecht, Utrecht, The Netherlands; 3 Department of Biomedical Engineering and Institute for Complex Molecular Systems, TU/e, Eindhoven, The Netherlands; 4 Xeltis BV, Eindhoven, The Netherlands

## Abstract

Vascular *in situ* tissue engineering encompasses a single-step approach with a wide adaptive potential and true off-the-shelf availability for vascular grafts. However, a synchronized balance between breakdown of the scaffold material and neo-tissue formation is essential. Chronic kidney disease (CKD) may influence this balance, lowering the usability of these grafts for vascular access in end-stage CKD patients on dialysis. We aimed to investigate the effects of CKD on *in vivo* scaffold breakdown and tissue formation in grafts made of electrospun, modular, supramolecular polycarbonate with ureido-pyrimidinone moieties (PC-UPy). We implanted PC-UPy aortic interposition grafts (n = 40) in a rat 5/6th nephrectomy model that mimics systemic conditions in human CKD patients. We studied patency, mechanical stability, extracellular matrix (ECM) components, total cellularity, vascular tissue formation, and vascular calcification in CKD and healthy rats at 2, 4, 8, and 12 weeks post-implantation. Our study shows successful *in vivo* application of a slow-degrading small-diameter vascular graft that supports adequate *in situ* vascular tissue formation. Despite systemic inflammation associated with CKD, no influence of CKD on patency (Sham: 95% vs CKD: 100%), mechanical stability, ECM formation (Sirius red^+^, Sham 16.5% vs CKD 25.0%–p:0.83), tissue composition, and immune cell infiltration was found. We did find a limited increase in vascular calcification at 12 weeks (Sham 0.08% vs CKD 0.80%—p:0.02) in grafts implanted in CKD animals. However, this was not associated with increased stiffness in the explants. Our findings suggest that disease-specific graft design may not be necessary for use in CKD patients on dialysis.

## INTRODUCTION

Patients with chronic kidney disease (CKD) and/or cardiovascular disease (CVD) regularly require vascular grafts to bypass or replace diseased blood vessels.^1,2^ Moreover, patients with CKD undergoing hemodialysis need vascular grafts to create vascular access for hemodialysis.^3^ Autologous veins are generally preferred as a source of vascular graft material due to high long-term patency and low risk of infection, but are often unavailable because of preexisting vascular pathology^4^ or show insufficient maturation.^5^ The current alternative, a synthetic vascular graft, is associated with poor long-term patency and higher risk for infection compared to autologous substitutes.^6,7^ The inert characteristics of non-degradable synthetic grafts result in compliance mismatch, deficient self-healing, and inadequate adaptive capacity, causing intimal hyperplasia (IH), calcification and/or thrombosis.^8^ Recent studies on vascular tissue engineering (TE), such as growing vascular grafts in a bioreactor^9^ or exploiting the foreign body response to create vascular constructs,^10,11^ have shown promising results. However, these approaches use lengthy, multistep creation processes with cell harvesting and maturation phases. The use of a completely degradable synthetic matrix, which simultaneously supports autologous tissue formation, provides a one-step *in situ* vascular TE approach with adaptive graft material characteristics and true off-the-shelf availability.

*In situ* vascular TE depends on a synchronized balance of breakdown of the scaffold material and neo-tissue formation through a process analogous to wound healing, including hemostasis, a controlled inflammatory response, cellular proliferation, and remodeling.^12^ By applying the body and its natural process of neo-tissue formation as a bioreactor allows for the transition from synthetic scaffold to autologous vascular conduit.^12–15^ Previous studies have shown that rapid scaffold degradation is associated with aneurysms and vascular deformations,^16^ whereas more retentive materials carry the potential risk of fibrosis and calcification.^17–19^ Conditions that affect neo-tissue formation and/or mechanical graft stability may, therefore, greatly influence the *in situ* vascular TE process. CKD is characterized by a disturbed haemostasis,^20,21^ thrombosis,^22,23^ a chronic pro-inflammatory and pro-fibrotic systemic environment^24,25^ with endothelial as well as vascular progenitor cell dysfunction,^26,27^ and increased calcification propensity.^28,29^ Whether and how CKD influences *in situ* vascular TE has not been previously assessed.

In the current study, we used a rat 5/6th nephrectomy model to mimic systemic conditions in human CKD patients^30,31^ in combination with an aortic interposition graft model to compare the patency, mechanical stability, and remodeling of a small-diameter vascular graft. We made use of supramolecular polycarbonate (PC) with ureido-pyrimidinone (UPy) moieties, which can be tailored with respect to their specific properties, e.g., biodegradability, (non)-cell adhesion, and mechanical properties (e.g., porosity and/or stiffness) to anticipate different disease-specific pathologic conditions.^32^ UPy-based polymers have demonstrated excellent biocompatibility in a range of models and applications,^33–35^ enabling tissue formation while the polymer gradually degrades. To improve our understanding of distinct phases of the wound healing process and potential undesired effects, such as vascular fibrosis and calcification, we monitored the grafts over a period of 2–12 weeks after implantation in CKD and healthy rats.

## RESULTS

### Survival and general characteristics

Animals were stratified based on their baseline uremic parameters (Table I). Eleven animals died due to acute kidney failure after CKD induction, but no further CKD-related mortality occurred at later time points. SNX resulted in significant increases in uremic parameters, and CKD animals reached the CKD threshold of 50 mg/24h proteinuria after 6–10 weeks. This was illustrated by elevated plasma creatinine (86.0 ± 5.6 vs 33.9 ± 1.9 *μ*mol/L; p < 0.0001), plasma urea (16.6 ± 0.6 vs 6.8 ± 0.2 mmol/L; p:<0.0001), proteinuria (82.2 ± 0.8 vs 4.2 ± 0.8 mg/24 h; p:<0.0001) and SBP (146 ± 5 vs 131 ± 3 mm Hg; p:0.0005), and a significant decrease in eGFR (629 ± 55 vs 2141 ± 82 *μ*l/min; p < 0.0001), while maintaining a similar weight (271 ± 4 vs 273 ± 5 g; p:0.7) [Table I—supplementary material Figs. 1(a)–1(d)]. At all time-points post-SNX, histological analysis of the remaining kidney of CKD rats revealed enlarged sclerotic glomeruli, dilated tubules, infiltration of inflammatory cells, regions of tubular necrosis and cast formation, and representative of CKD [supplementary material Fig. 1(e)].

**TABLE I.** Study characteristics. *SBP*: systolic blood pressure, *eGFR*: estimated glomerular filtration rate; data are represented as mean±SEM.

Uremic parameters	Sham	CKD	p-value^a^
Baseline				
Creatinine (*μ*mol/l)	30.6 ± 2.9	34.9 ± 4.0	0.39
Urea (mmol/l)	6.7 ± 0.3	6.2 ± 0.2	0.14
Proteinuria (mg/24h)	3.4 ± 0.7	4.6 ± 1.3	0.43
SBP (mmHg)	130.7 ± 3.5	132.3 ± 2.7	0.72
eGFR(*μ*l/min)	2071 ± 124	2015 ± 146	0.77
Weight (g)	227 ± 5	228 ± 3	0.88
Pre implantation				
Creatinine (*μ*mol/l)	32.9 ± 1.9	86.0 ± 5.6	<0.0001
Urea (mmol/l)	6.8 ± 0.2	16.6 ± 0.6	<0.0001
Proteinuria (mg/24h)	4.2 ± 0.8	82.2 ± 0.8	<0.0001
SBP (mmHg)	131.1 ± 3.1	145.9 ± 4.7	0.0005
eGFR (*μ*l/min)	2141 ± 81	629 ± 54	<0.0001
Weight (g)	273 ± 4	271 ± 3	0.71
Graft implantation				
Survival				
Survival implantation	21 (23)	22 (30)	
Patency grafts 24 h	21 (21)	20 (22)	
Patency end point	20 (21)	20 (20)	

^a^ Unpaired t-test.

Two Sham and 8 CKD animals died due to procedural technical failure (e.g., clamp leakage, overall blood loss), whereas 2 CKD animals died within 24 h post-implantation surgery. CKD animals overall showed lower overall resilience, in combination with higher blood loss, presumably due to slower hemostasis and higher blood pressure. The higher mortality of CKD as compared to Sham animals (27% vs 9%) with implantation is consistent with the severity of the 5/6th nephrectomy model and previous reports.^40^ At explantation, occlusion was visually observed in one graft in a Sham animal at 8 weeks. This animal remained asymptomatic during the experiment. All other grafts remained patent during follow-up.

### Circulatory immune cells

**FIG. 1.**
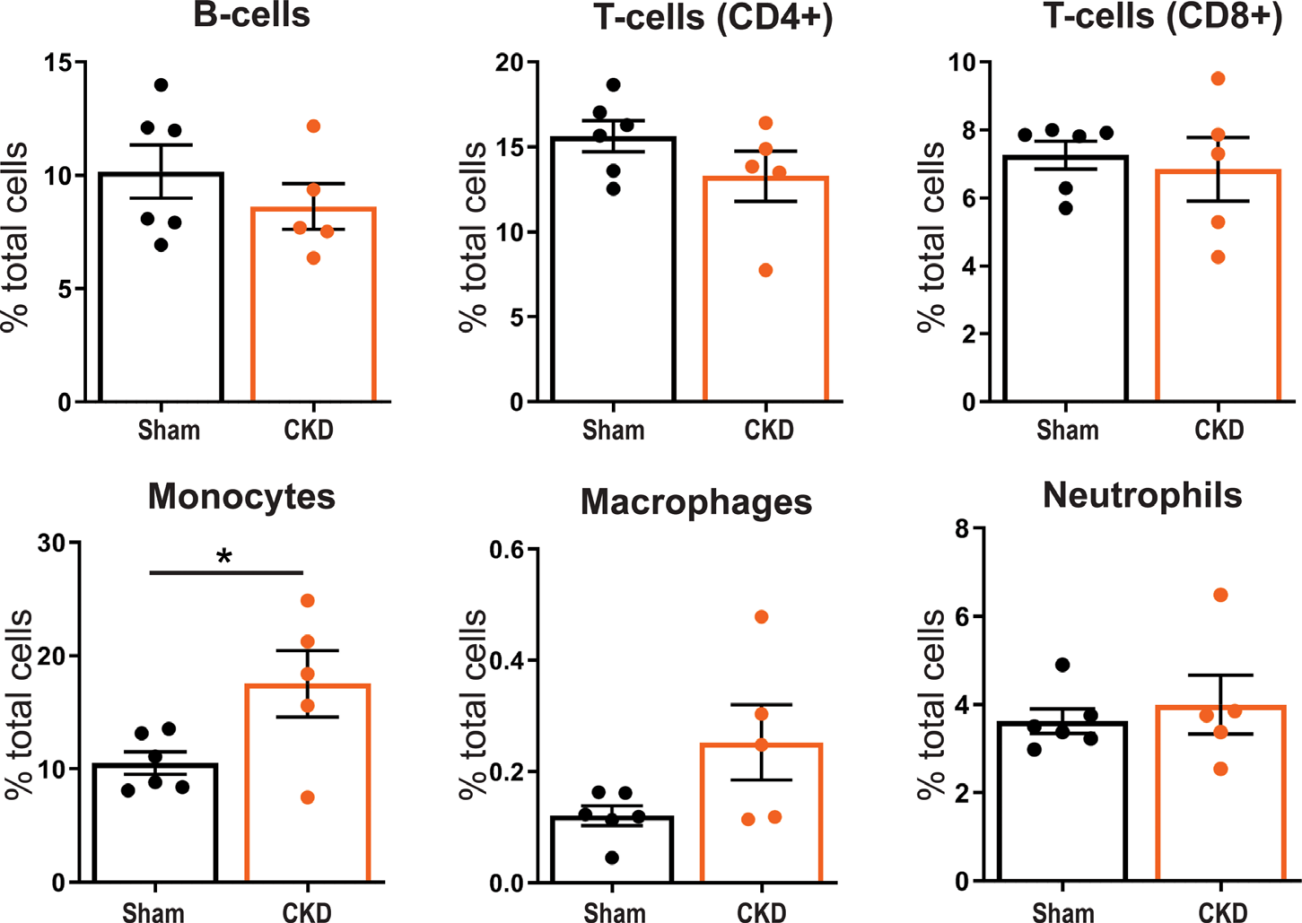
Percentage of total circulating immune cells. >10^4^ single cell events were acquired in whole blood of Sham (n = 6) and CKD (n = 5) animals and characterized by flow cytometry for RP1^+^ neutrophils, CD11b^+^ monocytes, CD45RA^+^ B-cells, CD4^+^ and CD8^+^ T-cells, and CD68^+^ macrophages. Mean ± SEM. ^*^p <0.05.

Flow cytometric analysis on blood 8 weeks post-SNX (supplementary material Fig. 2) showed that the monocyte fraction (CD11b^+^) was significantly (p:0.037) higher in CKD compared to Sham animals (17.5 ± 2.9% of total circulating leukocytes in CKD vs 10.5 ± 1.0% in Sham) (Fig. 1). We observed no significant differences in other major immune cell types, i.e., neutrophils (RP1^+^), B-cells (CD45RA^+^), CD4^+^ and CD8^+^ T-cells, and macrophages (CD68^+^ − p:0.069) between CKD and Sham.

### Extracellular matrix formation and cell infiltration in the grafts

**FIG. 2.**
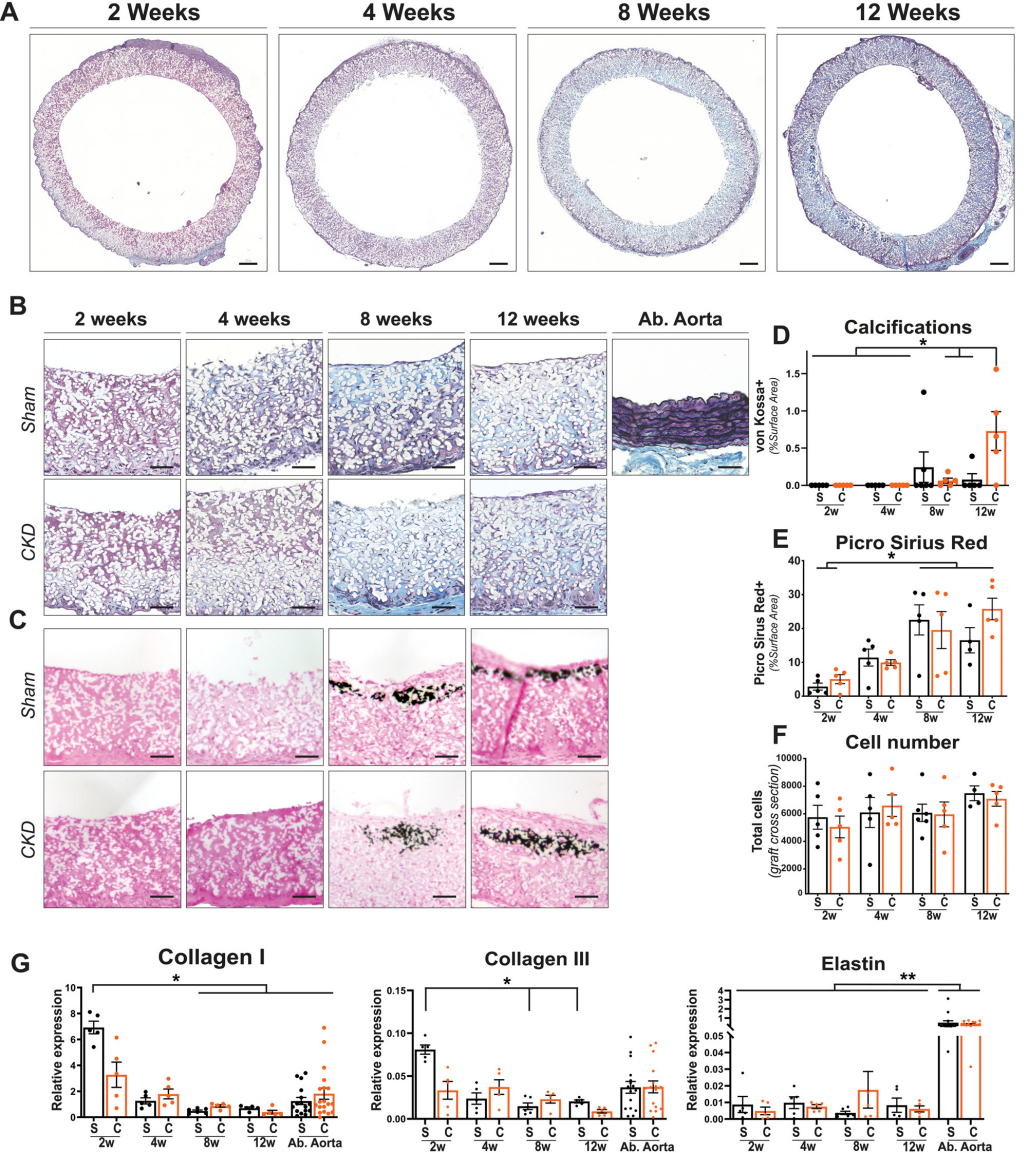
Histological evaluation on representative transverse sections. Explanted center part of the grafts at 2, 4, 8, and 12 weeks, stained with Masson's Trichrome Verhoeff (a). ECM formation and tissue buildup in sham and CKD animals over time, with an abdominal aorta as control. Visible are cytoplasma (pink), collagen (blue), elastin (black), and nuclei (dark blue). Electrospun fibers are visible in white. (b). Grafts stained with von Kossa and nuclear fast red to visualize calcification (black) in grafts explanted (c) and quantification thereof (d). Picro Sirius Red+ surface area showing an increase in ECM buildup over time (e). Total cell numbers in Sham(S) and CKD(C) graft (whole transverse sections) (f). Relative gene expression (2Δ^−CT^) of Collagen I, Collagen III, and Elastin **(**G) compared between Sham(S) and CKD(C) groups over time, with abdominal (Ab.) aorta from sham and CKD animals as controls. Scale bars represent 200(a) and 50 *μ*m (b) and (c). Mean±SEM. ^**^p < 0.01, ^*^p < 0.05.

A Masson's Trichrome Verhoeff stain indicated an increase in connective tissue deposited in the graft over time; however, the amount remained below native abdominal aorta level. Abdominal aorta controls also displayed mature elastin fibers, which were absent from explanted graft sections independent of time or disease state [Figs. 2(a) and 2(b)]. No differences in ECM composition between Sham and CKD animals were found in the abdominal controls (data not shown). Calcifications were found from 8 weeks onward in the media layer of the graft by von Kossa stain [Fig. 2(c)] and were significantly increased at 12 weeks in the CKD animals (Sham 0.08 ± 0.09% vs CKD 0.73 ± 0.26%; p: 0.04) [Fig. 2(d)]. Collagen content of the vascular grafts, as stained with Sirius Red (SR^+^), increased over time (positive surface area: Sham 2 weeks 2.8 ± 1.0% vs 12 weeks 16.5 ± 3.7%; p:0.004 – CKD 2 weeks 5.0 ± 1.3% vs 12 weeks 25.8 ± 3.2%; p:0.0018). No significant difference (p > 0.82) in SR^+^ area was detected between Sham and CKD graft sections at any time point [Fig. 2(e)—supplementary material Fig. 3]. The infiltrated number of cells inside the explants did not change significantly over time nor differed between Sham and CKD groups **[**Fig. 2(f)**]**. Gene expression of ECM markers Collagen I and III, typically deposited at an early stage in vascular *TE*, was significantly higher (p < 0.05) in Sham grafts at 2 weeks compared to later time points, returning to similar levels found in native abdominal aortas. In CKD animals, Collagen I and III expressions were not significantly different over time. No difference was observed in the expression of studied ECM-related genes between Sham and CKD animals in both graft and abdominal controls [Fig. 2(g)]. Significantly (P < 0.001) lower elastin gene expression was observed in explanted grafts compared to abdominal aorta controls, independent of time, and disease state [Fig. 2(g)].

### Immune cell presence in the grafts

**FIG. 3.**
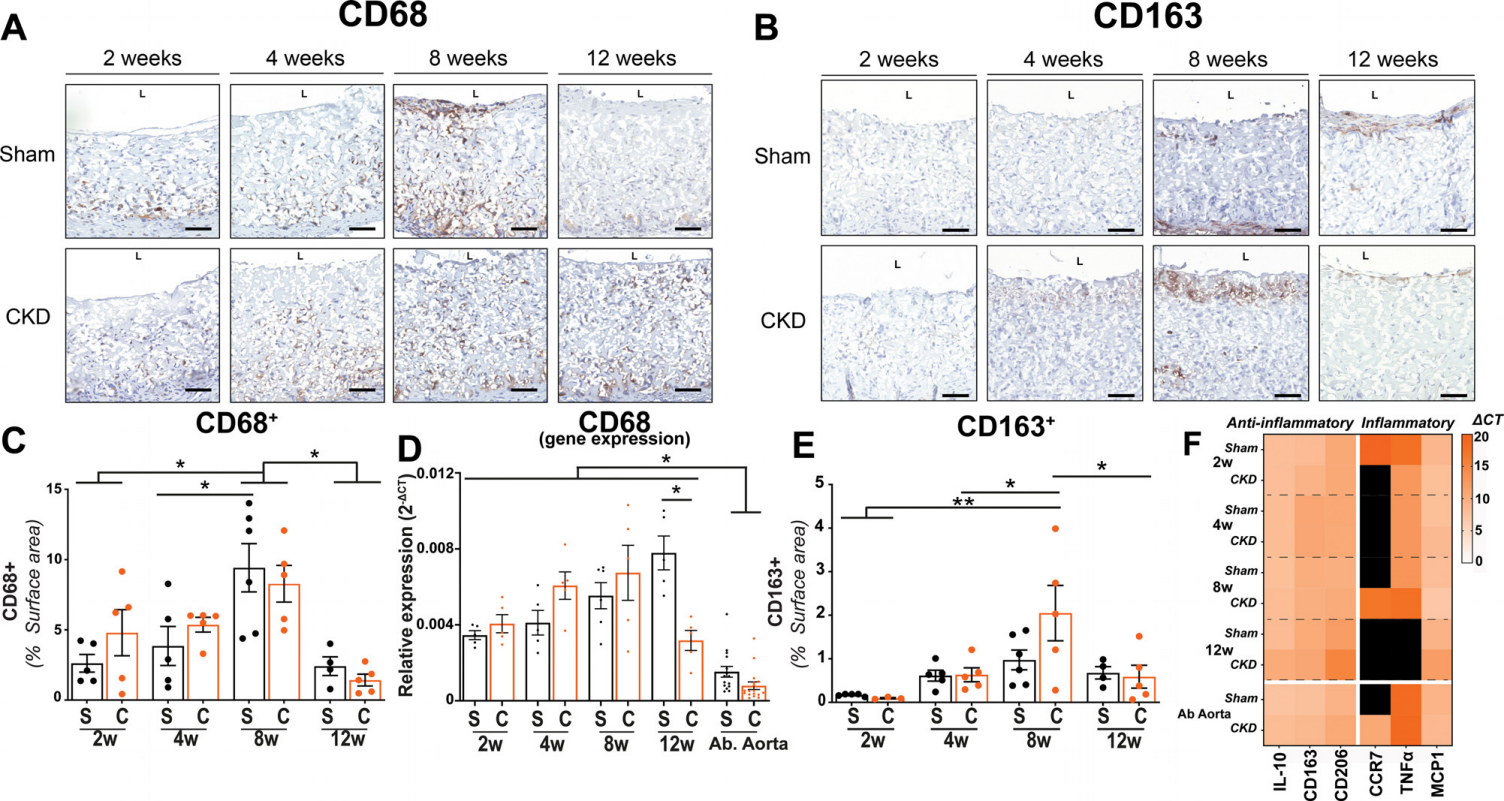
Histological evaluation of infiltration of immune cells. Transverse sections of the center part of the grafts, explanted at 2, 4, 8, and 12 weeks, stained for CD68^+^ (NovaRED) **(A)** and CD163^+^(NovaRED) (b), electrospun fibers are visible in white (a) and (b). Quantification of M1 macrophage marker CD68^+^ surface area percentage in Sham(s) and CKD(c) groups over time (c) and CD68 relative expression (2^−ΔCT^) with abdominal aortas (Ab Aorta) from Sham(s) and CKD(c) as controls (d). Quantification of M2 macrophage marker CD163^+^ surface percentage in Sham(s) and CKD(c) groups over time (e). Heatmap showing relative expression (2Δ^−CT^) of anti-(IL-10, CD163, CD206) and pro-inflammatory (CCR7, TNFα, MCP1) genes in graft sections in 2, 4, 8, and 12 week explant from sham and CKD animals. Abdominal aorta sections from Sham and CKD animals as controls. No detected expression in black (f). Scale bars represent 50 *μ*m. Mean±SEM. ^*^p < 0.05; ^**^p < 0.01.

The surface area of CD68^+^ positive macrophages in the explanted grafts of both Sham and CKD animals increased toward week 8 (Sham 2 weeks 2.6 ± 0.6%, 8 weeks 9.4 ± 1.7%; p:0.005—CKD 2 weeks 4.8 ± 1.6%vs 8 weeks 8.3 ± 1.3%; p:0.48) [Figs. 3(a) and 3(c)]. After week 8, the CD68^+^ surface area decreased significantly by 74% (p < 0.01) and 83% (p < 0.01), in Sham and CKD, respectively (Sham 12 weeks 2.4 ± 0.6%—CKD 12 weeks 1.42 ± 0.4%). At none of the time points did the CD68^+^ surface area differ (p:0.98) between Sham and CKD grafts. Our immunohistochemical findings corresponded with CD68 gene expression levels in the grafts [Fig. 3(d)], where CD68 gene expression levels at 12 weeks were still significantly higher in both groups compared to expression levels in native abdominal aortas (p < 0.001). The surface area of the anti-inflammatory M2-associated marker CD163^+^ cells in the grafts [Fig. 3(b)**]** was also highest at 8 weeks (Sham 0.9 ± 0.2%—CKD 2.0 ± 0.6%) and decreased toward week 12 (Sham: 0.7 ± 0.14%, CKD: 0.6 ± 0.3%) [Fig. 3(e)].

There were no significant differences (p > 0.1) between Sham and CKD grafts in expression of genes relevant for inflammatory and wound healing processes, including CCR7, TNF-a, and MCP-1 [Fig. 3(f)]. Expression of anti-inflammatory genes, such as CD163, CD206, and IL-10, was low (compared to reference genes) at every time point and did not change over time, showing similar levels as explanted native abdominal aortas. Additionally, no significant difference was detected between abdominal aorta controls from Sham and CKD animals for any of the (anti)inflammatory genes studied.

### Vascular cells in the grafts

**FIG. 4.**
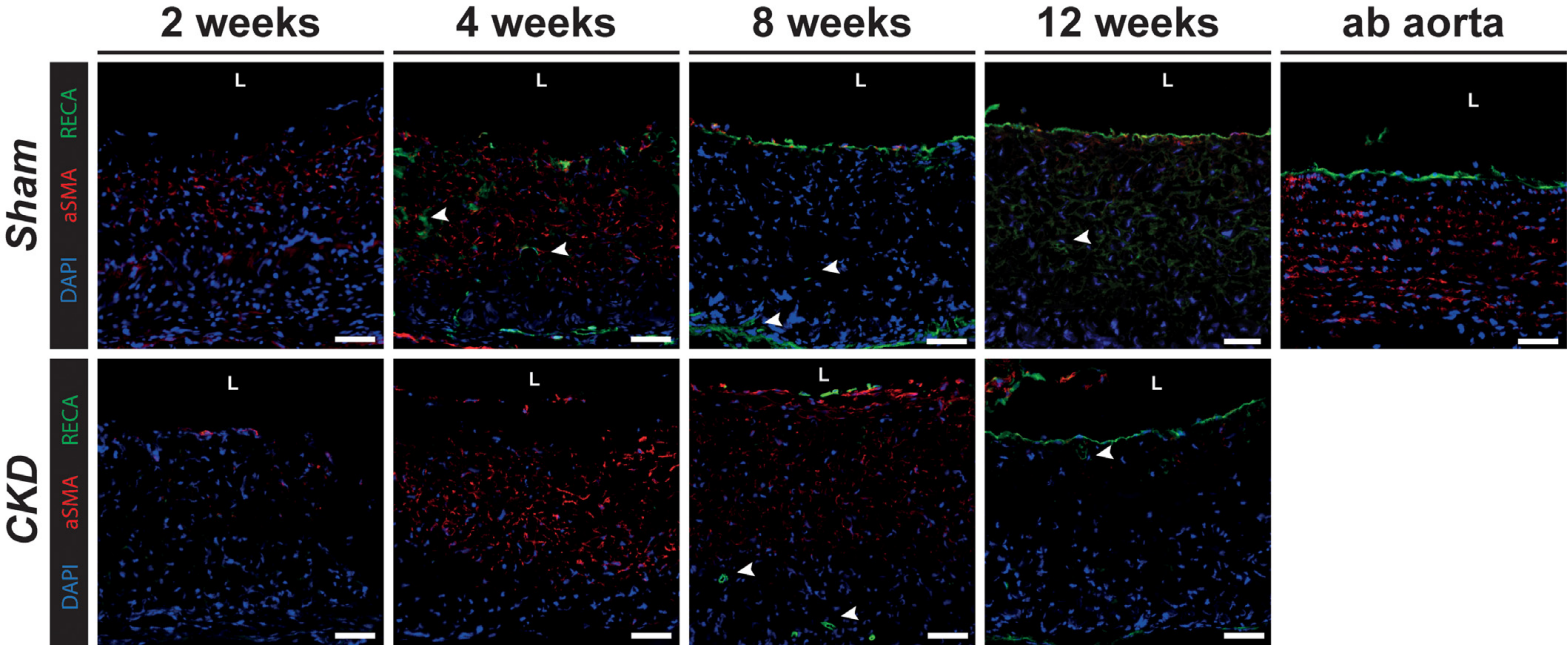
Vascular tissue formation. Grafts explanted from Sham and CKD animals at 2, 4, 8, and 12 weeks. Stained with rat endothelial marker, RECA (green) and smooth muscle cell marker, αSMA (red), with cell nuclei in blue (DAPI). Arrows indicate vascular infiltration. Lumen is indicated by L. Scale bars represent 50 *μ*m.

We observed an endothelial monolayer formation in both Sham as well as CKD grafts from 8 weeks onward, as visible in Fig. 4 by the presence of rat anti-endothelial cell antibody (RECA) positive lining on the luminal side and endothelial cell infiltration into the graft (indicated by white arrows). Alpha smooth muscle actin (αSMA) positive cells, frequently used as marker for myofibroblasts and smooth muscle cells, were primarily observed in the luminal half of the graft wall at the 4 and 8 week time points. They were not aligned, as was the case in the abdominal (ab) aorta that served as control.

### Mechanical properties and scaffold morphology

**FIG. 5.**
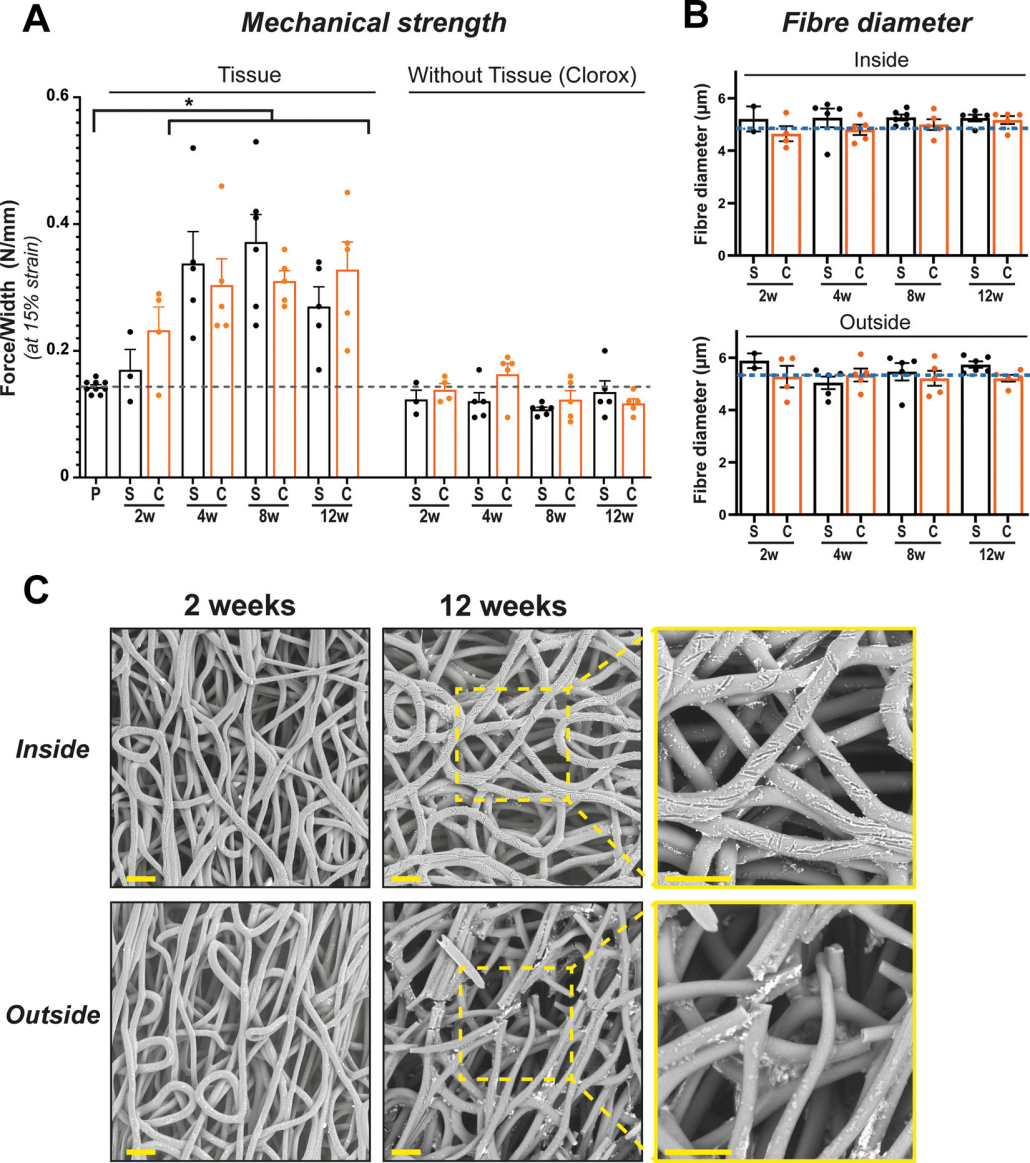
Mechanical properties and scaffold morphology. Mechanical strength increased over time due to tissue buildup and recedes to pristine (P) graft values after complete tissue removal by Clorox treatment (a). Stable fiber diameter, independent of Sham(S) or CKD(C) conditions (b), dotted lines represent the scaffold before implantation. Scanning electron microscopy photographs of fiber integrity over time show breakage in the fibers at 12 weeks on the outside of the graft, nicks in the fibers are observed at the luminal side (c). Scale bars represent 50 *μ*m. Mean ± SEM. ^*^p < 0.05.

From 4 weeks onward, explants showed a significant (p < 0.01) increase in mechanical strength (between 46% and 75%) compared to the pristine graft [Fig. 5(a)]. Stress–strain tests after Clorox treatment of the explants revealed that mechanical strength of the complete tissue-depleted scaffolds was not significantly reduced compared to pristine non-implanted grafts at all time points. No changes of fiber diameter were observed over time, both on the inner and outer layers of the graft [Fig. 5(b)]. Only at the 12-week time point, broken fibers were observed in the outer graft layer. We also observed shallow nicks in the fibers on the inner layer from week 2 onward [Fig. 5(c) and supplementary material Fig. 4).

## DISCUSSION

Our study shows that *in situ* small-diameter vascular TE graft implantation in a CKD rat model, using supramolecular PC-UPy scaffolds, provides high patency over a 12-week period and efficiently supports the build-up of autologous vascular tissue. CKD in this study did not affect hemostasis and thrombosis in grafts even without anti-coagulation therapy following implantation. Importantly, graft remodeling occurred without loss of mechanical strength of the graft, despite systemic uremic, and pro-inflammatory conditions associated with CKD. We did find increased vascular calcification in grafts in CKD vs Sham animals.

*In situ* vascular tissue engineering depends on balancing breakdown of the scaffold material and neo-tissue formation. As fast degrading scaffolds have previously been associated with vascular deformations and aneurysms,^16^ we chose for the use of a more retentive PC-UPy scaffold. The specific properties of the PC-UPy presented in this paper have been tuned to facilitate cell infiltration, degrade gradually, and maintain adequate functionality while synchronously being replaced by endogenous tissue.^41^ This approach has already been validated using functional graft implants in large animal models, which showed that the material needed to provide 3–6 months of mechanical support to allow sufficient time for neo-tissue to form and to replace functionality.^34,35^ *In vitro* degradation mechanisms are described in detail by Brugmans *et al.* who used enzymatic and oxidation models to demonstrate that, while conventional PCL (polycaprolactone) is more susceptible to hydrolysis, the PCL-UPy degrades predominately by oxidation.^41^ This was further elaborated by Marzi *et al.* who confirmed that *in vivo* degradation of PC-UPy (polycarbonate-UPy) is mainly driven by oxidative mechanisms as shown by Raman spectral signature.^35^ The exact rate of degradation and tissue ingrowth may vary depending on animal model and implant location.

Indeed, overall, our scaffold material showed limited degradation, with fiber breaks on scanning electron microscope images only visible after 12 weeks. Fiber breakage exclusively occurred on the outside of the graft, whereas we only observed shallow nicks in the fibers on the luminal side from week 2 onward, suggesting the involvement of tissue resident macrophages.^42^ Despite increased systemic macrophage activity^43^ and high reactive oxygen species production^44^ in CKD, which have been reported to affect scaffold degradation dynamics,^45^ we found no effect of CKD on scaffold integrity. We also observed no vascular deformations or aneurysms in healthy or CKD animals.

On the other hand, prolonged biomaterial presence has been suggested to induce chronic inflammation, frustrated phagocytosis through a foreign body reaction^46^ and the formation of a fibrotic network that is unable to remodel.^17,18^ In line, previous *in vivo* studies of slow-degrading biomaterials, such as polylactic acid (PLA), polyglycolic acid (PGA), and poly-ε-caprolactone (PCL), often showed a distinctive chronic inflammatory response and associated lower patency.^19,47–49^ However, in our study, we observed a diminishing inflammatory response after 8 weeks of implantation in both CKD and Sham groups.

Interestingly, recently Cramer *et al.* demonstrated that UPy-based materials are characterized by a pro-healing M2 macrophage-dominated response, in stark contrast to typical synthetic materials that trigger a pro-inflammatory M1 macrophage-dominated response.^50^ In our setting, CKD was not associated with increased presence of local inflammation and did not affect mechanical strength or vascular graft patency. From our experiments, we cannot exclude that CKD may have different effects when the balance between scaffold breakdown and neo-tissue formation is shifted in faster degrading grafts.

After graft implantation, we observed increased inflammatory cell numbers, gene expression profiles associated with pro- and anti-inflammatory cytokines, and subsequent neo-tissue formation in vascular grafts over time. Chronic systemic inflammation associated with CKD has been reported to adversely affect (chronic) wound healing;^51,52^ however, we observed no significant differences between CKD and Sham animals with respect to cellular infiltration or the dynamics of the inflammatory response and neo-tissue formation. We also observed no differences in cell proliferation, ECM build-up, and/or vascular tissue formation between CKD and Sham explants during the study. One previous study addressing *in situ* TE in diabetic disease conditions^53^ reported that one month after *in situ* vascular TE PCL-scaffold implantation, grafts in diabetic rats showed significantly decreased endothelial coverage and increased macrophage presence. In spite of reduced endothelial cell and progenitor function associated with CKD,^26^ we observed the presence of an endothelial layer from 8 weeks onward, in both CKD and Sham animals, which is known to be essential for long-term function of vascular tissue.^54^ Whether the discrepancy between our findings in CKD and the reported data in diabetes is due to differences between disease conditions or related to differences in scaffold materials is unclear. No aligned smooth muscle cells and mature elastin fibers were observed in our grafts, but proliferative cells were present and collagen fibers were actively deposited until week 8.

We did observe a limited increase in vascular calcification in the explanted grafts of CKD animals at 12 weeks. CKD is associated with vascular calcification, due to multiple risk factors that induce vascular smooth muscle cells to change into a chondrocyte or osteoblast-like cell.^55^ Calcification can affect vascular graft patency and increase the risk of all-cause and cardiovascular morbidity and mortality.^56,57^ In our study, calcification was limited to sections proximal to the anastomoses, suggesting a relation with mechanical stress and hemodynamic loading consistent with previous reports in TE heart valves.^58–60^ It has been shown that the continuous presence of foreign material in combination with the observed heightened systemic inflammation and metabolic changes caused by the CKD state may enhance calcification.^8^ Calcification levels observed in our study were not associated with an increase in stiffness in our explants. However, our data do not allow conclusions on the longer-term effects or complications in the graft. Notably, in a previous clinical study, a similar level of vascular calcification was not linked to an increase in complications and/or mortality.^61^ However, the use of modular materials, such as PC-UPy, in combination with enhanced mechanistic insight, may provide opportunities for modifications that inhibit formation of calcifications altogether.

Limited data are currently available on the effect of chronic disease states and systemic inflammation on tissue formation and vascular graft degradation in *in situ* vascular TE.^62^ A strength of our study is that we investigated *in situ* vascular TE graft implantation in a clinically relevant disease model. We employed the 5/6^th^ nephrectomy model in the rat, a well-known experimental model of progressive kidney disease, resembling CKD in humans. This rat model is characterized by elevated plasma creatinine and urea, proteinuria, hypertension, lower eGFR, and the enhanced presence of circulating inflammatory cells, reflecting a systemic, pro-inflammatory milieu associated with CKD.^63,64^ A limitation of our study is that the progressive nature of 5/6th nephrectomy induced CKD in rats does not allow follow-up of vascular tissue formation beyond three months. Additionally, we cannot exclude the influence of the higher post-implantation mortality in the CKD animals, due to the severe and progressive nature of the disease model, on our results. Future studies may employ less progressive models of CKD or combinations of CKD with other CVD risk factors to further elucidate long-term *in situ* TE vascular tissue formation. As we directly anastomosed our supramolecular grafts to the native aorta without applying shielding^65^ or a vascular loop,^66^ our model enables studying tissue formation irrespective of the origin of the infiltrating cells but limits us in answering questions on the source of infiltrating cells (e.g., circulation- or tissue-derived).

In conclusion, our study shows successful *in vivo* application of a biodegradable small-diameter vascular graft that supports adequate *in situ* vascular tissue formation. The graft retained its mechanical strength for three months without inducing a prolonged inflammatory response or causing graft occlusions, ruptures, fibrosis, or deformations. Importantly, CKD conditions did not significantly alter degradation dynamics, the wound healing process, neo-tissue formation, or endothelialization. Although vascular calcifications were more prominent in CKD, our findings suggest that there may be no need for disease-specific graft design for the clinical translation of these grafts to hemodialysis patients. These results should be confirmed in future studies using a true arterio-venous fistula in larger animal models that more closely resemble clinical practice.

## METHODS

### Experimental animals

**FIG. 6.**
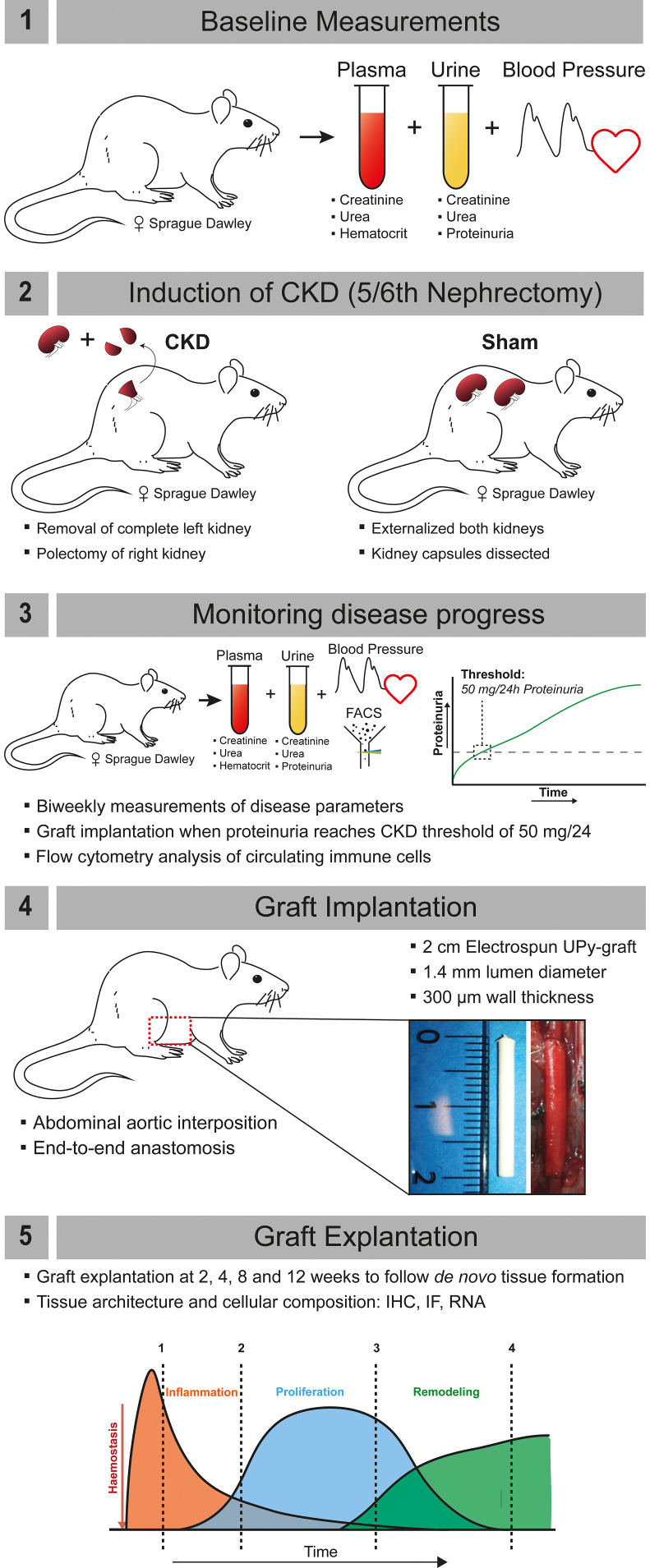
Schematic representation of the project work flow. (1) Baseline measurements were performed on plasma (creatinine, urea, hematocrit), urine (creatinine, urea, proteinuria), and systolic blood pressure (measured by tail cuff). (2) Induction of chronic kidney disease (CKD) by 5/6th nephrectomy; unilateral left side nephrectomy and polectomy of the right kidney. For sham animals, the kidneys were externalized and capsule was dissected. (3) CKD progression was monitored by measurement of plasma (creatinine, urea, and hematocrit), urine (creatinine, urea, proteinuria), and systolic blood pressure. When animals reached a proteinuria of 50 mg/24h, they were considered to have CKD, flow cytometry on circulatory immune cells was performed, and graft implantation occurred within 7 days. (4) 2 cm electrospun UPy-grafts with an inner diameter of 1.4 mm diameter were implanted as an abdominal aortic interposition graft by end-to-end anastomosis. (5) Grafts were explanted at 2, 4, 8, or 12 weeks to follow the process of *de novo* tissue formation. Tissue architecture and cellular composition were assessed by IHC, IF, and gene expression analysis.

Seventy-two female outbred Sprague Dawley (SD) rats (Envigo), aged 8 weeks and weighing approximately 200 g, were housed in groups up to 4, under standard climate-controlled conditions with *ad libitum* access to water and food. Animals were acclimatized for 7 days prior to start of the study. The study protocol was approved by the Animal Ethics Committee of the University of Utrecht (CCD-AVD1150020173344) and in agreement with the current Dutch law on animal experiments. Animals were stratified and allocated to Sham or CKD groups based on baseline measurements of proteinuria, creatinine, urea, and systolic blood pressure (SBP) (see Fig. 6 for a schematic representation and description of the experimental protocol).

### Model of chronic kidney disease in rats

CKD was induced by surgical 5/6th nephrectomy (SNX) as published,^36^ with minor adaptations. In short, in a single procedure, surgical removal of the whole left kidney and polectomy (2/3rd) of the right kidney was performed using retroperitoneal incisions. In Sham animals, both kidneys were externalized and the renal capsule was removed. Postoperative buprenorphine (0.03 mg/kg Temgesic) was given subcutaneously for a period of at least 36 h. Rats were weighed 3 times a week, 24-h urines, and blood samples were collected and the SBP was measured (Tail cuff sphygmomanometer–LE 5002 LETICA^®^) biweekly. To collect 24-h urines, rats were placed in metabolic cages without food for 24 h, but with free access to water with 2% glucose. Urine was collected in antibiotic/antimycotic solution (A5955; Sigma, St Louis, US) and stored at −20 °C. Blood samples were collected from the tail vein into EDTA Microtainers (BD #365974). Urinary protein levels were measured by Bradford Assay (Bio Rad). Plasma urea was determined by DiaSys Urea CT FS (DiaSys Diagnostic Systems). Plasma creatinine levels were determined by DiaSys Creatinine PAP FS kit (DiaSys Diagnostic Systems,). eGFR was calculated with a plasma creatinine- and urea-based equation specific for rats.^37^ We determined CKD as established once a threshold of proteinuria exceeding 50 mg/24 h was reached.

### Flow cytometry

After the proteinuria was reached, whole blood was collected from the tail vein of CKD animals and age matched Sham animals. Red blood cells were lysed with RBC Lysis/Fixation Solution (Biolegend #422401). Fixed cells were labeled with the following antibodies; anti-RP1-BV421 (1:100, BD Biosciences #743053), anti-CD45-BV510 (1:100, Becton Dickinson BV # 740140) anti-CD45RA-APC (1:100, Biolegend #202313), anti-CD3-Alexa488 (1:50, ITK Diagnostics #201406), anti-CD4-APC/Cy7 (1:100, ITK Diagnostics #201518), anti-CD8a-PE-Cy7 (1:100, Antibodychain #25–0084–82), anti-CD11b/c-Percp/Cy5.5 (1:100, ITK Diagnostics #201820), and anti-CD68-PE (1:100, Antibodychain #MA516653). Antibody labeling was performed for 30 min at 4 °C in FACS buffer (10% FCS, 0,1% sodium azide in PBS). Subsequently the cells were washed twice using PBS. Flow cytometric analyses (≥10^4^ events acquired in the “single cell” gate) were performed using a Becton Dickinson FACSCanto II. Gating (supplementary material, Fig. 2) was performed with fluorescence minus one and unstained controls.

### Scaffold fabrication

Tubular porous grafts were constructed by electrospinning polycarbonate-UPy (PC-UPy) using established protocols in our labs.^38,39^ The grafts with an inner diameter of 1.4 ± 0.1 mm, a wall thickness of 300 ± 15 *μ*m, and a length of 20 ± 0.5 mm were produced using electrospinning with a specified fiber diameter of 5 ± 1 *μ*m, as characterized by scanning electron microscope. The PC-UPy is based on a telechelic polycarbonate soft block with relatively low (<5 kDa) molecular weight that is chain extended with UPy-units in the hard block. More details on the synthesis can be found in Mes *et al.*^39^

### Vascular graft implantation

Within one week after reaching the CKD threshold of 50 mg/24 h proteinuria (Fig. 6), the scaffolds were implanted as an end-to-end interposition graft in the abdominal aorta. Grafts were explanted after 2, 4, 8, or 12 weeks to follow the different stages of tissue formation. Age-matched Sham animals were used as controls. For the graft implantation, animals were anesthetized using isoflurane gas (4% induction, 2.5% maintenance, and O_2_). A midline laparotomy was performed, and the abdominal viscera were externalized in damp cloth to expose the retroperitoneal cavity including the abdominal aorta and the inferior vena cava. The aorta was dissected from the inferior vena cava and surrounding tissue, and any collateral branches from the aorta were coagulated. Twenty units of heparin were given intravenously through the tail vein before clamping the segment of the abdominal aorta between the renal arteries and the aortic bifurcation with microvascular clamps (ST ABB-2). The interposition graft (length 2 cm) was implanted using an end-to-end anastomosis at both ends in the abdominal aorta using interrupted sutures (9.0 ETHILON – Ethicon 2809 G). After removing the vascular clamps, patency was visually confirmed by verifying the pulsatile flow in the aorta distal to the graft. Animals in which flow could not be reconstituted were excluded. The abdomen was closed by suturing the muscle layers with continuous stitches (4.0 Vicryl—Ethicon V392H) and the skin with continuous intracutaneous stitches (5.0 Vicryl—Ethicon V303H). Postoperative buprenorphine (0.03 mg/kg Temgesic) was given subcutaneously for a period of at least 36 hours. No anti-coagulation was given after surgery. Patency was checked during the experiment by verifying pulse in the tail during SBP measurements (Tail cuff sphygmomanometer - LE 5002 LETICA^®^), hindleg function retainment and visual and histological inspection at explantation. At termination, animals were anesthetized using isoflurane and sacrificed by exsanguination (cardiac puncture). Grafts and relevant organs were dissected from surrounding tissue, cut into segments, and stored for subsequent analysis.

### Immunohistochemistry

Kidney remnants and explanted grafts were fixed in 4% PFA and embedded into paraffin for sectioning (3 μm thickness) before staining. Hematoxylin and Eosin, Masson's trichrome Verhoeff, Periodic Acid Schiff (PAS), von Kossa and Picro Sirius Red (SR) stainings were performed using standard techniques. Graft sections were stained (60 min at RT) using CD68 (pan-macrophage marker, ab31630, 1:250; Abcam,) and CD163 (M2 macrophage marker; ab182422, 1:500; Abcam) and visualized with NovaRED (SK-4800, Vector Laboratories). Whole cross sections. were scanned and digitized with the AxioScan M200682 scanner (Carl Zeiss Microimaging Inc.) and analyzed by two blinded scorers with ImageJ open software. The positive surface area was measured by selecting the whole tissue cross-section as area of interest, separating the area stained with NovaRED by using the color deconvolution plugin and setting a threshold. We then calculated the % positive surface area by dividing the separated NovaRED positive area by the total tissue area ×100. Cell nuclei were separated by water shedding and subsequently counted using ImageJ open software.

### Immunofluorescence

Explant segments were subsequently fixed in 4% PFA with 15% sucrose, followed by 15% sucrose solution without fixative for 24 h, 30% sucrose solution for 24 h, and embedded in OCT Tissue-Tek (Sakura #4583). Consecutive 5 *μ*m sections were stained for rat specific endothelial marker RECA (mouse anti-rat RECA, MCA970R, 1:100, 60 min Serotec), with secondary Brightvision αMo-poly-HRP (DPVM55HRP, 30 min, Immunologic) followed by tyramide-FITC (NEL701001KT, 10 min, 1:50 in amplification buffer) and direct Cy3-conjugated smooth muscle marker; αSMA (αSMA-Cy3, C6198, 60 min, 1:500; Sigma). Whole sections were imaged with a Leica DMi8 THUNDER Imager.

### Scanning electron microscopy

Upon explantation, segments of the graft used for scanning electron microscopy were exposed for 10 min with a 5% sodium hypochlorite (Clorox) solution to digest the tissue completely (e.g., extracellular matrix and cellular debris) on top of and in between the polymer fibers to allow scanning electron microscope visualization of the polymer. The protocol has been validated to ensure adequate tissue removal without triggering degradation of the polymer itself. After that samples were dehydrated and were put on the scanning electron microscope with either the abluminal or luminal surface to visualize (degradation-related) changes to the graft microstructure. Images were recorded using a Phenom desktop scanning electron microscope (Thermo Fisher Scientific). The accompanying FiberMetric software was used to determine fiber diameter as the average of 10 measurements in a representative image at 3500× magnification.

### Mechanical analysis

For mechanical characterization, a 10 mm long explanted graft segment was retrieved and longitudinally cut in half, after which one half was untreated and the other half treated for 10 min with 5% sodium hypochlorite solution (Clorox) to dissolve tissue. Not only decellularizing the explant but also completely removing the tissue (e.g., extracellular matrix and cellular debris) on top of and in between the polymer fibers allows for singular assessment of the mechanical strength of the remaining polymer. The treated and untreated strips were subjected to tensile testing in axial direction. Force at 15% strain (normalized by sample width) was derived as a relative measure for stiffness.

### RNA isolation, cDNA synthesis, and qPCR

A 1 mm cross section of graft and abdominal aorta was snap frozen with liquid nitrogen. These snap frozen sections were halved, and RNA was extracted with Trizol reagent (Invitrogen, CA, USA) until the aqueous phase step, followed by the use of RNA extraction columns (Bioline #BIO-52073). RNA (200 ng) was reverse transcribed (25 °C–10 min; 42 °C–15 min; and 85 °C–5 min) using the Bioline SensiFAST™ cDNA Synthesis Kit (Bioline #BIO-65054). A total of 4 ng cDNA were used for quantitative analysis (two-step RT-PCR, 8.5 min at 95 °C; [38 × 15 s at 95 °C; 45 s at 60–63.5 °C]; 1 min at 95 °C) of rat genes (supplementary material Table 1) with FastStart Universal SYBR Green (Roche #4913914001), followed by a melting curve. B2M and GAPDH were used as standard housekeeping genes. The relative mRNA expression levels were determined using the ΔCT method.

### Statistics

Prior to commencing the animal experiments, a power calculation was performed with G*power, t-test with two groups one-way based on cell infiltration data in similar *in vivo* experiments performed in our group. Data are presented as means ± standard error of the mean (SEM) and considered statistically significant for p < 0.05. T-tests and 2 way-ANOVA for repeated measures (Tukey post-hoc) were used appropriate with GraphPad Prism 9 software.

## SUPPLEMENTARY MATERIAL

See the supplementary material for the complete primer list used for qPCR, kidney disease progression of CKD vs Sham animals, the flow cytometry gating strategy, the histology images of the Picro Sirius Red staining, and for the scanning electron microscopy photographs of fiber integrity of all time points (luminal and adventitial).

## Data Availability

The data that support the findings of this study are available from the corresponding author upon reasonable request.
